# Lyme arthritis in Southern Norway - an endemic area for Lyme Borreliosis

**DOI:** 10.1186/1471-2334-14-185

**Published:** 2014-04-05

**Authors:** Glenn Haugeberg, Inger Johanne W Hansen, Tone Skarpaas, Sølvi Noraas, Vivian Kjelland

**Affiliations:** 1 Department of Rheumatology, Hospital of Southern Norway Trust, Servicebox 416, 4604 Kristiansand, Norway; 2 Faculty of Health and Sport Sciences, University of Agder, Kristiansand, Norway; 3 Department of medical microbiology, Hospital of Southern Norway Trust, Kristiansand, Norway; 4 Department of Engineering and Science, University of Agder, Kristiansand, Norway; 5 Research Unit, Hospital of Southern Norway Trust, Kristiansand, Norway

## Abstract

**Background:**

Despite Southern Norway is an endemic area for Lyme borreliosis there is a lack of data on Lyme arthritis (LA). In the literature controversies exist if acute LA can develop into chronic arthritis. Our objective was to identify and characterize patients with LA in Southern Norway and explore disease course after antibiotic treatment.

**Methods:**

Patients aged 20 years or older with arthritis and a positive serology for Borrelia burgdorferi infection (IgG and/or IgM) suspected of having LA were consecutively recruited either from general practitioners or from hospital departments.

**Results:**

From January 2007 to December 2010 a total of 27 patients were assessed. Mean (range) age was 56 years (41–80) and mean symptom duration prior to inclusion was 11.2 weeks (1 day – 2 years). Definite LA was diagnosed in 16 patients, probable LA in 5 patients and 6 patients were concluded to have other arthritis disorders. Among the 21 LA patients 20 had mono-arthritis (knee 18, ankle 2) and 1 had polyarthritis.

All LA patients responded favourable to antibiotic treatment and none of the patients developed chronic arthritis after long term follow up, not even in LA patients who had intraarticular glucocorticosteroid (GC) injection prior to antibiotic treatment.

**Conclusions:**

Our data shows that LA in Southern Norway is a benign disease which successfully can be treated with antibiotics even in patients treated with GC prior to antibiotics.

## Background

Lyme borreliosis is caused by the spirochete *Borrelia burgdorferi* sensu lato (Bb) and is the most common tick-borne disease in the northern hemisphere [1]. The coastal part of Southern Norway is known as an endemic area for Lyme borreliosis [2]. In this area, *Ixodes ricinus* ticks are prevalent and Bb is found in about 25% of the ticks [3] and IgG antibodies against Bb is found in approximately 20% of the adult population [4].

The main attention in Norway on Bb infections has been on Lyme neuroborreliosis [5-8]. Despite arthritis being a well-known manifestation of Bb infection, there is a lack of data on Lyme arthritis (LA) in Norway. In the literature there are controversies whether LA after antibiotic treatment can develop into chronic arthritis [9,10]. Our clinical experience has been that LA is successfully treated with antibiotics without causing chronic arthritis even in patients treated with GC prior to antibiotic treatment.

Thus our aim was prospectively to identify and characterize patients with LA in Southern Norway in a long term follow up study and to illuminate treatment response to antibiotic in these patients.

## Methods

### Recruitment of patients and collection of clinical data

Patients suspected of having LA were consecutively recruited from Southern Norway. The patients were assessed at the outpatient rheumatology clinic at the Hospital of Southern Norway Trust in Kristiansand. In 2007 the hospital covered a population of 268 461 inhabitants (195 492 inhabitants aged above 20 years).

Patients aged ≥ 18 years with acute arthritis with a positive Bb laboratory test suspected to have LA were consecutively assessed and included. A positive Bb test was defined by the presence of serum IgG and/or IgM antibodies against Bb and/or by a positive polymerase chain reaction (PCR) test for Bb in synovial fluid. The patients were either recruited from general practitioners who referred arthritis patients with a positive serology or from our own rheumatology department.

Data on demographic and clinical characteristics were collected. Clinical data collection included history of previous tick bite(s) and erythema migrans, duration of arthritis, joint(s) affected, and previous anti-inflammatory treatment including intra-articular injections of glucocorticosteroids (GC). Microscopy and white blood cell count in synovial fluid was also performed when joint fluid was available. Data for erythrocyte sedimentation rate (ESR), C-reactive protein (CRP), was also collected.

Treatment and treatment effect during follow up was recorded. The included patients were followed in the outpatient clinic by experienced rheumatologists. All assessed patients were also informed to take direct contact with the rheumatology outpatient clinic in case of recurrent joint symptoms.

### Definition of definite and probable Lyme arthritis

Definite LA was defined by the presence of arthritis excluding other arthritis disorders diagnosed by experienced rheumatologists (IJWH, GH) and the presence of serum IgG antibodies against Bb and/or a positive PCR test against Bb in synovial fluid. Probable LA was defined as presence of arthritis and detected serum IgG antibodies against Bb and/or a positive PCR test against Bb, however other diagnosis of arthritis could not be definitely excluded as the causative factor. No LA was concluded when arthritis was explained by other obvious rheumatic joint diseases e.g. osteoarthritis and psoriatic arthritis, despite the presence of positive Bb serology.

### Detection of antibodies to Borrelia burgdorferi sensu lato

Sera were examined for Bb IgM and IgG antibodies by an EIA using B. garinii (PKo strain) as antigen (Enzygnost Borreliosis, Dade Behring, Marburg GmbH, Germany) (EnzygnostM, EnzygnostG). From 18.11.2008 the IgG test with added VlsE was used (Enzygnost Lyme link VlsE IgG).

### Detection of Borrelia burgdorferi sensu lato

DNA was isolated from joint fluid using (Magnapur). Purified DNA was stored at -20°C. DNA extracts were examined for Bb spirochetes by using a real-time PCR with probe (FAM-5’-TTCGGTACTAACTTTTAGTTAA-MGBNFQ) and primers (forward and reverse primer sequences were 5’GCTGTAAACGATGCACACTTGGT and 5’GGCGGCACACTTAACACGTTAG, respectively) specific for a section of the 16S ribosomal RNA (rRNA) gene [11]. Real-time PCR was performed as previously described [3]. Briefly, real-time PCR was performed using iCycler/MyIQTM (Bio-Rad). The 25 μl PCR mixture included 1X ready-to-use reaction mixture (TaqMan Universal PCR Master Mix, Applied Biosystems Inc.) which contains reaction buffer, Taq DNA polymerase, deoxynucleoside triphosphate and MgCl2. The final concentration of the primers and probe were 1.125 μM and 0.25 μM, respectively. Finally, 5 μl of template DNA was added. The PCR conditions were as follows: 50°C for 2 min and 95°C for 10 min, followed by 50 cycles of 95°C for 15 s and 63°C for 60 s. Differentiation of Bb strains was done by a species specific, single run, real-time PCR based on the protein HBb (hbb) gene sequence [12] as previously described [3]. The melting points of the amplicons generated from the unknown samples and from known Bb species were compared for genotyping. In cases where a clear identification could not be made, genotyping was done by direct sequencing of the chromosome located rrs (16S) – rrlA (23S) intergenic spacer (IGS) [13]. PCR and sequencing were performed as previously described [3]. Negative and positive samples were included in all runs. Precautions were taken at all steps of analysis to avoid contamination of samples.

### Statistics

Descriptive statistics was applied and analysed by SPSS for windows, version 16. Continuous variables were presented as mean with range in parentheses and categorical variables as numbers and percentages (%).

### Ethics

The study was approved by the ethical committee for Southern Norway Helse Sør (REK S-06474a). All patients gave written informed consent before inclusion.

## Results

From January 2007 to December 2010 a total of 27 patients suspected to have LA were assessed at the outpatient clinic. Mean age was 56 years (41–80). Mean symptom duration prior to assessment at the outpatient rheumatology clinic was 11.2 weeks (1 day – 2 years).

Definite LA was diagnosed in 16 patients (10 men, 6 females), probable LA in 5 patients (3 men and 2 females) and no LA in 6 patients (2 rheumatoid arthritis, 2 psoriatic arthritis and 2 knee osteoarthritis). In the group of patients with probable LA 2 patients also had a severe osteoarthritis, 2 patients were HLAB27 positive and peripheral spondyloarthritis could not be excluded, and 1 patient had a severe osteochondral lesion of the affected ankle joint.

Mean follow up time from inclusion to last visit at the outpatient clinic for all 27 patients was 70 weeks (range 15–292) and for patients with definite and probable LA 54 weeks (15–152). All medical records from the 27 patients were finally reviewed in September 2013 and none of the patients were after antibiotic treatment found to have recurrent arthritis which could be attributed to LA.

A history of prior erythema migrans was reported in 7 of the 27 included patients, 4 in the definite group, 1 in the probable group and 2 in the group diagnosed not to have LA. A history of prior tick-bite was reported in 11/16 patients in the definite and 3/5 in the probable LA group and in all 6 patients in the non LA group.

In Table 1 demographic and disease characteristics are shown for each single patient with definite and probable LA. Among the 16 patients with definite arthritis all had mono-arthritis (knee 15 and ankle 1) and among the 5 patients with probable LA 4 had mono-arthritis (knee 3 and ankle 1) and 1 polyarthritis (both knees and several small joints in the left foot).

**Table 1:** Characteristics of 16 patients with definite Lyme arthritis and 5 patients with probable Lyme arthritis

**Definite**												
**Age (years)**	**Gender**	**History of prior tick bite**	**History of prior EM**	**Duration of arthritis (weeks)**	**Joint affected**	Minor flares after antibiotic treatment*	**ESR (mm/h)**	**CRP (mg/dl)**	**Serum Bb IgG % of cut off at baseline**	**Serum Bb IgM positive test at baseline**	**PCR positive test for Bb in joint fluid**	**Intraarticular GC before antibiotic therapy**
46	Male	Yes	No	3	Left knee	None	32	13	2423	Yes	Yes	Yes
54	Male	Yes	No	1	Left knee	None	55	89	700	No	Yes	Yes
47	Female	Yes	No	1	Right knee	One	45	1	1350	Yes	Yes	No
68	Male	Yes	No	104	Left knee	Two	13	51	1550	Yes	Yes	Yes
65	Male	Yes	No	1	Left knee	Two	48	129	1200	+/-	Yes	Yes
70	Female	Yes	No	7	Right knee	None	13	1	1500	No	No	No
50	Female	Yes	Yes	3	Left knee	None	9	2	2273	Yes	No	No
49	Male	Yes	Yes	1	Left ankle	None	37	74	2400	No	No	No
50	Male	Yes	Yes	1	Right knee	None	23	33	2335	Yes	NA	Yes
59	Male	Yes	No	3	Left knee	One	32	13	2423	Yes	No	No
54	Female	No	No	1.5	Left knee	None	9	1	1350	No	Yes	Yes
55	Male	No	No	12	Right knee	One	38	24	1350	No	Yes	Yes
66	Male	No	No	2.5	Right knee	None		67	NA	NA	Yes	No
51	Female	Yes	Yes	12	Right knee	None	20	1	1450	No	Yes	No
50	Female	No	No	5	Left knee	None	15	30	NA	NA	Yes	No
47	Male	No	No	2	Left knee	None	29	93	1300	+/-	Yes	No
**Probable**												
**Age (years)**	**Gender**	**History of prior tick bite**	**History of prior EM**	**Duration of arthritis (weeks)**	**Joint affected**	Minor flares after antibiotic treatment*	**ESR (mm/h)**	**CRP (mg/dl)**	**Serum Bb IgG % of cut off at baseline**	**Serum Bb IgM positive test at baseline**	**PCR positive test for Bb in** **joint** **fluid**	**Intraarticular GC before antibiotic therapy**
41	Male	Yes	No	0	Left ankle	None	8	53	2100	No	No	No
80	Male	No	No	3	Left knee	†	35	3	2550	No	No	No
53	Female	Yes	No	2	Both knees and several small joints in left foot (MTP and PIP-joints)	One	68	90	2500	No	No	No
50	Male	No	No	0	Right knee	None	68	132	1750	Yes	No	No
56	Female	Yes	Yes	4	Left knee	†	15	1	1300	Yes	No	No

EM: Erythema migrans. ESR: Erythrocyte sedimentation rate. CRP: C-reactive protein. Bb: Borrelia burgdorferi. IgG: Immunoglobulin type G. IgM: Immunoglobulin type M. PCR: Polymerase chain reaction. GC: Glucocorticosteroids. NA: Not assessed. MTP: Metatarsophalangeal joints. PIP: Proximal interphalangeal joints.

*Defined as episodes of arthritis symptoms the patients reported the first weeks/months after antibiotic treatment.

+/- Non conclusive test.

†Concomitant osteoarthritis with recurrent episodes of joint effusion.

At time of inclusion for the 21 LA patients, the mean ESR was 31 mm/h (8–68), mean CRP was 43 mg/dl (1–132) and mean white cell count in synovial fluid available from 14 patients was 18 G/L (0–101).

The Bb IgG and IgM results for each of the 21 LA patients are displayed in Table 1. All LA patients who had been tested (19/21) had high level of serum IgG antibodies to Bb (mean 1800% of cut off, range 700–2550). The 2 patients who were not tested for antibodies to Bb were included due to detection of Bb in synovial fluid by PCR and a clinic suggestive of LA.

Bb IgM antibody tests were positive in 6/16 patients in the definite group and 2/5 in the probable LA group. Further 2 patients had an inconclusive serum IgM Bb test. Among the 15 tested patients in the definite LA group a positive borrelia PCR test in synovial fluid was found in 11 patients. Among the 5 patients in the probable group and among the 4 patients in the no LA group tested, none had a positive Bb PCR test.

Only one patient in the definite LA group with age 68 years with disease duration of 104 weeks (see Table 1) had several recurrent arthritis flairs before diagnosed as LA. The other patients with a suggestive history suggestive of LA had a disease duration that ranged from a few days to 12 weeks.

Among the LA patients 19 were treated with doxycycline for 2–8 weeks and one patient was treated with cloxacilline and cefotaxime for 14 days each. This one patient was initially suspected to have septic arthritis. The one patient with the long disease duration of 104 weeks described above was first treated for 3 weeks with doxycycline and thereafter for 4 weeks with doxycycline due to a more prolonged clinical arthritis.

In the 16 patients with definite LA, 11 patients had no flares, 3 patients had one and 2 patients had 2 flares. These flares, apart from in one patient, were episodes of arthritis symptoms the patients reported the first weeks and months after antibiotic treatment. In one patient a more significant flare occurred 3 months after his first treatment. He was then treated with a second course of doxycycline for 4 weeks, thereafter no flare occurred. None of the patients developed chronic arthritis or recurrent arthritis after long term follow up. Among the 5 patients with probable LA, 2 had no flares, 1 had one flare and 2 got recurrent episodes of joint effusion. These two patients also had severe osteoarthritis.

Seven LA patients had intraarticular GC injection prior to and one during antibiotic treatment. All patients responded favourable to GC injection however all relapsed. Conversely, none relapsed after antibiotic treatment.

Based on the present study, the estimated annual incidence rate for LA in individuals age 20 years or older in the four years period was 2.7 per 100 000 inhabitants in the region.

## Discussion

In our study we confirm that mono-arthritis in the knee is the most common manifestation of LA as already recognized in the first description of LA in 1977 [14]. As also shown in our patient cohort, other large joints such as the ankle joint may also be involved, whereas upper limb large joints and small joins were rarely involved [14,15]. Further the described recurrent nature of the LA disease was also seen in our study in 6 out of 16 patients with definite LA and 3 out of 5 patients with probable LA [14].

We only included adult individuals, which is a limitation of our study as LA has been reported to be more frequent in children than in adults [14].

In Norway, all systemic clinical manifestations of Lyme borreliosis (but not erythema migrans) are notifiable to the Norwegian Institute of Public Health. In Norway in the 10 year period from 1995 to 2004 a total of 1 506 cases (annually ranging from 120 to 253) were reported with Lyme borreliosis other than erythema migrans [2]. In this report the highest incidence was found in children aged between 5 and 9 years. Lyme neuroborreliosis was the most common reported clinical manifestation (71%), followed by arthritis/arthralgia (22%) and acrodermatitis chronica atrophicans (5%). In the same geographic area of Southern Norway where our study was carried out, an annual incidence rate of Lyme neuroborreliosis of 10 per 100 000 adult inhabitants has been reported [8]. Estimated annual incidence rates for LA in adults was in our study 2.7 per 100 000 inhabitants. However, the numbers of adult patients with Lyme borreliosis from Southern Norway reported to the Norwegian Institute of Public Health was 63 in 2007, 53 in 2008, 29 in 2009 and 23 in 2010, yielding an annual incidence ranging from approximately 12 to 32 per 100 000 adult inhabitants, with an average annual incidence of 21/100 000 [16]. Thus, the number of identified patients with LA in our study is probably an underestimation of the real number of patients in this geographical area. In a study estimating the incidence of Lyme borreliosis in the county of Westchester, New York in US the incidence was estimated to be several fold higher than suggested by the reporting system [17]. In our geographical area patients with LA most likely have been treated by general practitioners, and some patients may have been treated by the two private practicing rheumatologists working in the hospital area. Another reason may be that patients only had a short episode of arthritis and that signs and symptoms then spontaneously disappeared and thus were not referred. Minor transient attacks with arthritis were also observed in the cohort of untreated LA patients described by Steere and colleges [15].

The clinical signs and symptoms seen in LA are not diagnostically specific for the disease. Prior to the availability of laboratory tests, patients with LA were frequently diagnosed e.g. as reactive arthritis and RA in adults and as pauciarticular juvenile arthritis in children [15]. The use of laboratory tests detecting antibodies against Bb has improved sensitivity and specificity diagnosing LA. PCR techniques have also become available to detect borrelial DNA directly in synovial fluid. However the concomitant presence of antibodies against Bb and other inflammatory joint diseases may be a challenge diagnosing these patients. This was also seen in our patient cohort of 27 patients where 16 patients were found to have definite and 5 patients a probable LA. In the later group 2 patients also had severe knee osteoarthritis, 2 patients were HLAB-27 positive and one patient also developed a severe osteochondral lesion of the affected ankle joint.

The best description of the natural clinical evolution of LA in patients with erythema migrans was described by Steere and colleges in the 1980s [15]. In this cohort of patients with erythema migrans not treated with antibiotics, who were followed longitudinally for a mean duration of 6 years, approximately 60% developed arthritis. No arthritis was observed in 20% and 18% had only brief episodes of joint and muscular pain. In the arthritis group 51% had one episode or began to have intermittent attacks of frank arthritis whereas 11% of the patients developed chronic synovitis later in the illness [15]. Acrodermatitis chronica atrophicans is a well known chronic manifestation of Lyme borreliosis. Apart from this manifestation there are major controversies whether there is a chronic form of Lyme disease and if there is a subgroup of patients who require long term antibiotic treatment beyond 1–2 months [9,18]. All attempts to illuminate this possibility have not revealed any objective evidence of such a hypothesized link between an on-going, active infection with Bb, and chronic clinical signs and symptoms [9,19]. None of our LA patients with mean follow up of 54 weeks developed chronic arthritis and all patients were successfully treated with antibiotics. In the literature however, 10% of the LA patients has been reported to develop chronic arthritis [10]. This may be explained by other chronic arthritis diseases or by an actual intractable chronic arthritis not responding to antibiotic treatment.

The administration of intraarticular GC prior to antibiotic treatment in LA is not recommended. In a study by Bentas and colleges they found that patients who had received intraarticular GC prior to antibiotic treatment required significantly more courses of antibiotic treatment and the time required for disappearance of the arthritis was longer [20]. In our study all LA patients treated with intraarticular GC injections responded favourably with temporary relief of clinical signs and symptoms, however all flared. But, after antibiotic treatment none of the patients flared or developed chronic arthritis.

## Conclusions

LA in Southern Norway seems to be less frequent than Lyme neuroborreliosis and is a rather benign disease which is successfully treated with antibiotics even in patients treated with GC prior to antibiotics. Thus the challenge is to be aware of the disease, to diagnose LA and treat the patients properly with recommended antibiotics.

## Competing interests

The author(s) declare that they have no competing interests.

## Author’s contributions

GH participated in data collection, analyzing the data, drafting the manuscript and was the principle investigator. IJWH participated in data collection, analyzing the data and drafting the manuscript. TS and SN performed all Bb laboratory analysis and contributed analysing data and drafting the manuscript. VK performed all PCR Bb laboratory analysis and contributed analysing data and drafting the manuscript. All authors read and approved the final manuscript.

## Pre-publication history

The pre-publication history for this paper can be accessed here:

http://www.biomedcentral.com/1471-2334/14/185/prepub
